# Report of the Committee on Preliminary Education, Appointed under the 5th Resolution Adopted by the National Medical Convention of May, 1846

**Published:** 1847-09

**Authors:** 


### RECORD OF MEDICAL SCIENCE.

*Report of the Committee on Preliminary Education, appointed under the 5th Resolution adopted by the National Medical Convention of May*, 1846.—The duty entrusted to the Committee was "To report on the standard of acquirements which should be exacted of young men, before being received as students of medicine."

Before attempting to perform this duty, the Committee thought it desirable to ascertain, by inquiries addressed to the medical schools and distinguished practitioners of medicine throughout the Union, the sentiments and practice of the profession on this interesting subject. They, accordingly, prepared a circular, the object of which was to ascertain the views of the various Medical Schools in regard to the standard of preliminary education which it is proposed to exact of young men about to commence the study of medicine; and also to invite any suggestions they might think proper to make. Copies of this circular were sent to the (then) thirty-six Medical Schools of the United States, and official replies have been received from six of this number; namely, the Medical Department of the University of Pennsylvania, the College of Physicians and Surgeons in the City of New York, the Medical Institution of Yale College, Ohio Medical College, Albany Medical College, and the Medical Faculty of Dartmouth College. These institutions are desirous of having a standard established, and the Committee do not doubt that they, as well as others, will cheerfully co-operate with the Convention and the profession in this desirable reform.

Another circular was addressed to distinguished medical practitioners residing in all the States and Territories of the Union, soliciting replies to the following four questions.

1. Is it the custom of your State or neighborhood to require of young men, who desire to study medicine, any particular amount of preliminary education?

2. If so, what are the details of your standard?

3. By what authority, whether of a State Medical Society, local Association, or common consent, is this standard established?

4. What may be considered the general sentiment of the profession in your vicinity, in regard to the establishment of such a standard as is contemplated in the 5th resolution of the National Medical Convention of May, 1846?

The Committee have been favoured with very full and explicit answers to this circular from thirty-nine gentlemen, representing twenty-one States of the Union. The replies which have been received to the first three questions, establish the fact, not only that there is no uniform standard of preparatory education exacted of medical students throughout the United States, but that there is no general rule adopted in any particular state or district, which has been authorized or recommended by Medical Societies or other official bodies, or established by common consent and custom. The whole subject is left to private preceptors, many of whom recommend, and a few exact, an elevated standard, while others leave it to the discretion of the students themselves, or their parents. But all the letters of the practitioners indicate, without an exception, the cheering fact, that the profession is alive to the want of a standard, is desirous that one should be established by the Convention, and is willing to sustain it.

The fourth question was designed to elicit the general sentiment of the profession in regard to the nature and extent of the preliminary education which should be required of medical students. On this subject, the correspondence shows a considerable diversity of opinion; and the standard of different writers varies from a "common school" education up to the highest collegiate attainments. Some advocate a different scale in different sections of the country, while a majority is in favour of having the standard uniform throughout the Union. And, notwithstanding the individual differences of ideas, there is a sufficient general concurrence in the views of the writers to enable the Committee to recommend a scale which is chiefly based upon their united suggestions.

Your Committee are aware of the difficulties the Convention would discover in fixing the standard of preliminary education for medical students as high as would be desirable. Entirely destitute of the means of legal compulsion, and depending for success, as the Convention must, solely upon the force of professional and pubiic opinion, nothing could be hoped from a standard above the circumstances of the country and the times. The existing evil can be reached only by the concurrent action of private medical preceptors, and the medical schools of the country. The chief responsibility rests with the preceptors; and to them the Convention should make its strongest appeal; but at the same time they should be encouraged and sustained by the co-operation of the schools, without which, indeed, the efforts of preceptors could be but partially successful.

The object to which the Committee has directed its labours, it is believed, can be best effected by the Convention in the following way:—

*1st.* By establishing a uniform standard of preliminary education for medical students, which shall be of a moderate character—in the first instance, too low, rather than too high—and yet of such extent as will insure both the knowledge and the mental discipline necessary to those who would enter a profession full of labour and responsibility, without excluding meritorious young men of limited means and opportunities.

*2d.* By earnestly recommending every medical preceptor to exact this standard of every young man, before admitting him into his office; and having exacted it, to grant him a written certificate to that effect, specifying also the period of his admission into the preceptor's office, as a proper warrant and credential for the student, when about entering a medical college.

*3d.* By requesting all the medical colleges of the country to require such a certificate of every student applying for matriculation; and, in publishing their annual list of graduates, to accompany the name of the graduate with the name and residence of his preceptor, the name of the latter being clearly and distinctly presented as certifying to the qualification of preliminary education.

These ideas the Committee have put into the form of distinct resolutions, which they append to their report, submitting both for the consideration of the Convention, and, if it think proper, its adoption.

1*st.* *Resolved*, That this Convention earnestly recommends to members of the medical profession throughout the United States, to satisfy themselves, either by personal inquiry or written certificate of competent persons, before receiving young men into their offices as students, that they are of good moral character, and that they have acquired a good English education, a knowledge of Natural Philosophy and the Elementary Mathematieal Sciences, including Geometry and Algebra; and such an acquaintance, at least, with the Latin and Greek languages, as will enable them to appreciate the technical language of medicine, and read and write prescriptions.

2*d.* *Resolved*, That this Convention also recommends to the members of the Medical profession of the United States, when they have satisfied themselves that a young man possesses the qualifications specified in the preceding resolution, to give him a written certificate, stating that fact, and recording also the date of his admission as a medical student, to be carried with him as a warrant for his reception into the medical college in which he may intend to pursue his studies.

3d. *Resolved*, That all the medical colleges in the United States be, and they are hereby recommended and requested to require such a certificate of every student of medicine applying for matriculation; and when publishing their annual list of graduates, to accompany the name of the graduate with the name and residence of his preceptor, the name of the latter being clearly and distinctly presented as certifying to the qualification of preliminary education.

Signed by JAMES COUPER, L. P. BUSH, JAMES W. THOMSON, ALDEN MARCH, WASHINGTON L. ATLEE, D. T. BRAINARD, *Committee.*

